# Starvation of low-density lipoprotein-derived cholesterol induces bradyzoite conversion in *Toxoplasma gondii*

**DOI:** 10.1186/1756-3305-7-248

**Published:** 2014-05-29

**Authors:** Fumiaki Ihara, Yoshifumi Nishikawa

**Affiliations:** 1 National Research Center for Protozoan Diseases, Obihiro University of Agriculture and Veterinary Medicine, Inada-cho, Obihiro Hokkaido 080-8555, Japan

**Keywords:** *Toxoplasma gondii*, Cholesterol, LDL, Squalene synthase, Bradyzoite

## Abstract

**Background:**

Lacking enzymes for sterol synthesis, the intracellular protozoan *Toxoplasma gondii* scavenges cholesterol from host cells to multiply. *T. gondii* has a complex life cycle consisting of two asexual stages; the proliferative stage (tachyzoite), and the latent stage characterized by tissue cysts (bradyzoite). *In vitro*, bradyzoite development can be induced by mimicking host immune response stressors through treatment with IFN-γ, heat shock, nitric oxide, and high pH. However, the extent to which host nutrients contribute to stage conversion in *T. gondii* is unknown. In this study, we examined the impact of host cholesterol levels on stage conversion in this parasite.

**Methods:**

Growth of *T. gondii* tachyzoites (ME49 strain) was investigated in Chinese hamster ovary (CHO) cells using various concentrations of low-density lipoprotein (LDL), oleic acid, or glucose. Squalestatin, which is an inhibitor of squalene synthase and is, therefore, an inhibitor of sterol synthesis, was used to treat the CHO cells. Tachyzoite to bradyzoite conversion rates were analyzed by indirect fluorescent antibody tests.

**Results:**

Parasite growth was significantly enhanced by addition of exogenous LDL, whereas no such enhancement occurred with oleic acids or glucose. In ME49, growth inhibition from squalestatin treatment was not obvious. Although growth of the RH strain was unaffected by squalestatin in the presence of lipoprotein, in its absence growth of this strain was suppressed. The frequency of BAG1-positive vacuoles in ME49 increased under lipoprotein-free conditions. However, addition of exogenous LDL did not increase tachyzoite to bradyzoite conversion in this strain. Furthermore, treatment with squalestatin did not enhance stage conversion.

**Conclusion:**

Our results suggest that LDL-derived cholesterol levels play a crucial role in bradyzoite conversion in *T. gondii*.

## Background

The obligate intracellular parasite *Toxoplasma gondii* replicates inside a host cell in a specialized nonfusogenic vacuole known as the parasitophorous vacuole (PV) [1]. Successful replication of *T. gondii* within the PV requires considerable amounts of the specific lipids needed for membrane biogenesis. *T. gondii* autonomously synthesizes phospholipids, but can also readily scavenge lipid precursors from host cells [2,3]. Previously, *T. gondii* was shown to be auxotrophic for low-density lipoprotein (LDL)-derived cholesterol and that interfering with host cholesterol acquisition by *T. gondii* impairs its growth [4]. Although *T. gondii* cannot synthesize sterol, sterol esterification has nevertheless been detected in this parasite [5]. Cholesterol ester (CE) synthetic enzymes, CE synthesis [2,5], and acyl-CoA: cholesterol acyltransferase (ACAT) enzymatic activity have been described in *T. gondii*[5]. Regarding other esterification reactions in *T. gondii*, one study indicated that triacylglycerol (TAG) formation in this parasite occurs through an acyl-CoA: diacylglycerol acyltransferase (TgDGAT)-mediated pathway [6]. *T. gondii* can acquire lipids from the host and modify them to TAG and CE by TgDGAT1 and TgACAT1, respectively, resulting in the formation of lipid bodies in the parasite [5,6]. Furthermore, *T. gondii* infection triggers lipid body accumulation in host cells [7,8].

*Toxoplasma* disseminates within a host primarily through interconversion between two asexual stages, tachyzoites and bradyzoites. Differentiation of fast-replicating tachyzoites into dormant bradyzoite-stage parasites is pivotal to tissue cyst formation, which allows life-long persistence of viable parasites in the host. Tissue cysts containing bradyzoites are found in many host organs, but appear to preferentially develop in neural and muscular tissue [9]. The early events of parasite stage conversion are thought to be of critical importance, where expression of tachyzoite-specific genes is switched off and bradyzoite-specific genes are upregulated [10]. *In vitro* methods that stimulate tachyzoite to bradyzoite interconversion are well-established. Bradyzoite development can be induced by mimicking the stress of the host immune response through treatment with interferon-gamma (IFN-γ), high temperature (43°C), nitric oxide, high pH (pH = 8.1), and/or mitochondrial inhibitors [11-15]. Additionally, specific organ or cell factors can trigger high levels of stage conversion and tissue cyst formation [16]. Although *T. gondii* has a highly clonal population structure comprising three wide-spread and similar lineages, referred to as types I, II and III, representative strains of these clonal lineages show equal ability to differentiate into bradyzoites *in vitro*, with the exception of the RH strain (type I) [17,18]. However, the contribution that host cholesterol metabolism makes to *T. gondii* stage conversion is unknown. Therefore, we hypothesized that impairing host cholesterol levels would induce bradyzoite conversion and affect parasite survival. In the present study, to confirm this hypothesis, we examined the effects of host cholesterol on intracellular growth and bradyzoite conversion in *T. gondii*.

## Methods

### Parasites and cell cultures

The *Toxoplasma gondii* RH and ME49 strains used in this study were maintained in human foreskin fibroblast (HFF) cells cultured in Dulbecco’s modified Eagle medium (DMEM, Sigma, St. Louis, MO) supplemented with 10% heat-inactivated fetal bovine serum (FBS). Chinese hamster ovary (CHO) cells were cultured in Ham’s F-12 medium (Gibco BRL, Grand Island, NY) supplemented with 10% heat-inactivated FBS. To purify tachyzoites, the parasites and host-cell debris were washed with cold PBS, and the final pellets were resuspended in cold medium and then passed through a 27-gauge needle and a 5.0-μm-pore filter (Millipore, Bedford, MA).

### Reagents

Squalestatin and oleic acid were obtained from Sigma (St. Louis, MO). Human LDL (density 1.019–1.063 g/mL) was purchased from Biomedical Technologies Inc. (Stoughton, MA). Lipoprotein-deficient serum (LPDS) was prepared by ultracentrifugation of FBS after its density was increased to 1.215 g/mL with potassium bromide [4]. The cholesterol concentration in the LPDS was estimated to be under the minimum level detectable by a commercial detection kit (Cholesterol E-test Wako, Wako Pure Chemical Industries).

### Parasite growth analyses

CHO cells (1 × 10^6^) infected with *T. gondii* tachyzoites (5 × 10^4^) were cultured in 0.5 mL of medium in 24-well plates. After incubation for 44 h at 37°C, [5,6-^3^H] uracil (Moravek Biochemicals, Brea, CA) was added to the plates at 1 μCi/well and the cell mixtures were incubated for a further 2 h at 37°C. After fixation with 10% trichloroacetic acid for 30 min, the cell mixtures were incubated with 0.2 N NaOH for 30 min at 37°C. Radioactivity incorporated into the parasites was measured using a beta counter (Perkin-Elmer, Boston, MA).

### Indirect fluorescent antibody test (IFAT)

Parasite conversion was investigated using coverslips with confluent CHO or HFF cells infected with the ME49 strain of *T. gondii.* The coverslips were collected 72 h after parasite inoculation, washed twice with PBS containing 1 mM CaCl_2_ and 1 mM MgCl_2_ (PBS++) and then fixed with 3% paraformaldehyde in PBS++. After washing twice with PBS++, the cells were permeabilized with 0.3% Triton X-100 in PBS++ for 5 min at room temperature. After washing, the coverslips were incubated with 3% bovine serum albumin (BSA) in PBS++ at room temperature for 30 min. The coverslips were incubated with BAG1 rabbit anti-serum and an anti-SAG1 mouse monoclonal antibody (clone TP3; Advanced ImmunoChemical Inc., Long Beach, CA) diluted at 1:100 in 3% BSA in PBS++ for 1 h at room temperature. After washing three times with PBS++, the coverslips were incubated with Alexa Fluor 488-conjugated goat anti-rabbit IgG and Alexa Fluor 594-conjugated goat anti-mouse IgG (Sigma, St. Louis, MO) diluted at 1:1000 in 3% BSA in PBS++ for 1 h at room temperature and then washed again with PBS++. The coverslips were placed on a glass slide coated with Mowiol (Calbiochem, San Diego, CA), and the slides were examined using a fluorescence microscope (Nikon, Tokyo, Japan). A total of 100 infected cells were counted and the percentage of BAG1-positive vacuoles was determined.

## Results

### Growth differences associated with LDL, fatty acids, and glucose

**Figure 1:**
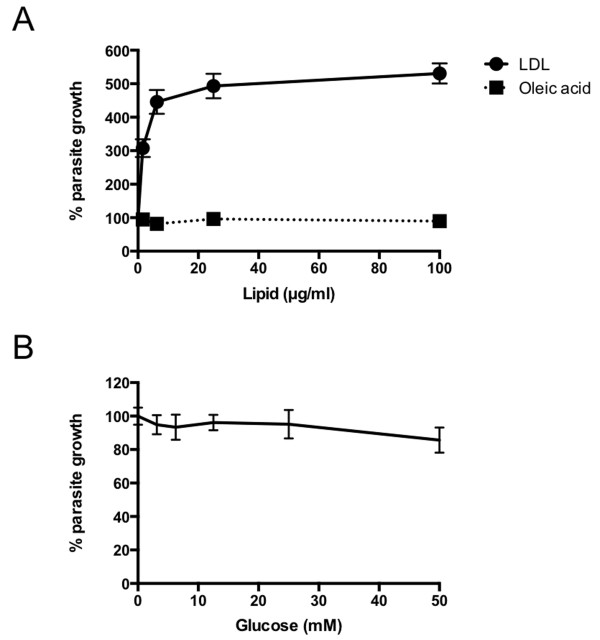
**Parasite growth in the presence of LDL, fatty acids, or glucose.** Uracil incorporation was assayed using CHO cells infected with the ME49 strain of *T. gondii* for 20 h in medium containing 5% LPDS treated with LDL or oleic acid at the concentrations indicated **(A)**, or in glucose-free medium containing 10% FBS treated with glucose at the concentrations indicated **(B)**. Data are expressed as a percentage relative to the control (incubation without lipid or glucose), which was taken as 100% ± the standard deviation (*n* = 4).

To investigate the factors important for parasite growth, we investigated the growth of *T. gondii* tachyzoites (ME49 strain) in CHO cells (Figure 1). Under lipoprotein free conditions, parasite growth was significantly enhanced by exogenous LDL (Figure 1A). However, neither exogenous oleic acids nor glucose stimulated parasite growth (Figure 1A, B).

### Effect of host cholesterol on parasite growth

**Figure 2:**
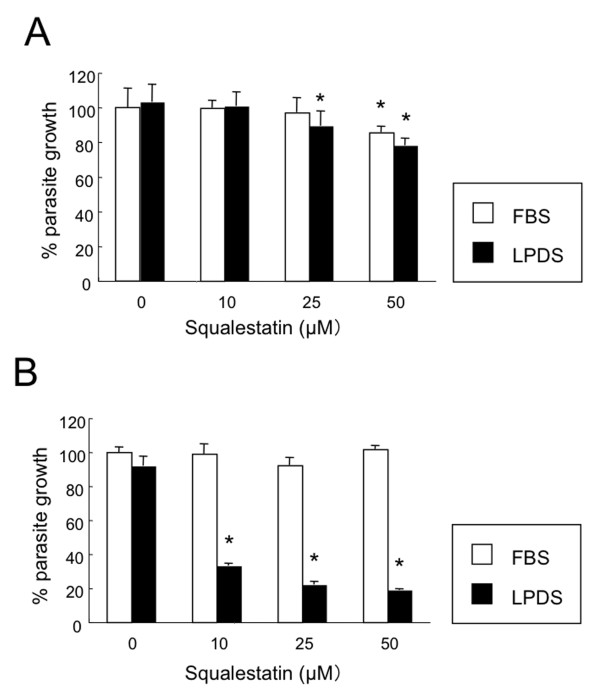
**Squalestatin treatment and parasite growth.** Uracil incorporation was assayed using CHO cells infected with the ME49 strain **(A)** or RH strain **(B)** of *T. gondii* for 44 h in medium containing 5% LPDS or 5% FBS treated with squalestatin at the concentrations indicated. Data are expressed as a percentage relative to that of the control (incubation in the presence of 5% LPDS without squalestatin), which was taken as 100% ± the standard deviation of triplicate samples. Statistical analysis of the data was conducted using a one-way ANOVA followed by Tukey’s multiple comparison tests. (*) Values of *P* < 0.01 were considered statistically significant when compared with incubation without squalestatin.

We focused on the effect of host cholesterol levels on *T. gondii* growth in CHO cells because cholesterol is one of the major components of LDL (Figure 1). No significant differences were observed when the parasites were grown in medium containing 5% FBS or 5% LPDS (Figure 2). Squalestatin, which is a squalene synthase inhibitor and is, therefore, an inhibitor of sterol synthesis, was used to inhibit cholesterol synthesis in CHO cells. Growth inhibition of ME49 by squalestatin was not obvious in medium containing 5% FBS or 5% LPDS (Figure 2A). However, in medium containing 5% FBS, squalestatin treatment did not affect the growth of the RH strain, while the growth of this strain was suppressed by squalestatin treatment in medium containing 5% LPDS (Figure 2B). This result indicates that host cholesterol starvation affected growth in the RH strain.

### Effect of host cholesterol starvation on bradyzoite conversion

**Figure 3:**
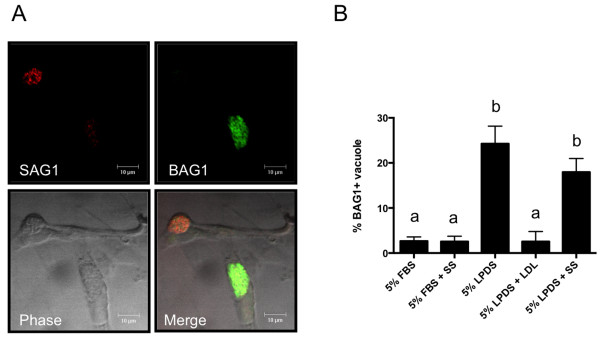
**IFAT analysis of BAG1 expression in parasite vacuoles.** CHO cells were infected with *T. gondii* ME49. After 72 h, the parasite-infected cells were subjected to IFAT analyses. **(A)** Infected CHO cells were stained with a SAG1 antibody (tachyzoite specific marker) and a BAG1 antibody (bradyzoite specific marker). Scale bar: 10 μm. **(B)** Percentage of vacuoles expressing BAG1. Data represent the mean percentage of BAG-positive vacuoles from one hundred vacuoles (from triplicate samples). Statistical analysis of the data was conducted using a one-way ANOVA followed by Tukey’s multiple comparison tests. Different symbols indicate statistically significant differences (*P* < 0.01). SS, 50 μM squalestatin.

Because the ME49 strain was resistant to host-cholesterol starvation in CHO cells, we speculated that variations in host cholesterol levels would influence bradyzoite conversion. To investigate whether host cholesterol induced tachyzoite to bradyzoite stage-conversion in *T. gondii*, IFAT analyses were performed using the bradyzoite-specific marker, BAG1, and tachyzoite specific marker, SAG1 (Figure 3). The frequency of BAG1 expression in the parasites increased in medium containing 5% LPDS compared with medium containing 5% FBS. However, addition of exogenous LDL did not enhance bradyzoite conversion in medium containing 5% LPDS. Furthermore, treatment with squalestatin did not enhance stage conversion in medium containing 5% LPDS or 5% FBS. These results indicate that low levels of LDL-derived host cholesterol trigger bradyzoite conversion.

## Discussion

Developmental stage conversion in *T. gondii* is a complicated process. Tachyzoite to bradyzoite transformation has been shown *in vitro* through induction of stress by host cell treatment with IFN-γ or mitochondrial inhibitors [11,12], alkaline pH, or high temperature [14,16]. Thus, exogenous stressors appear to influence the developmental differentiation of *T. gondii*. In this study, we investigated how host cholesterol levels affected stage conversion in *T. gondii*. Suppression of parasite growth is important for bradyzoite conversion [12]. Here, LDL-derived host cholesterol was one nutrient found to be involved in *T. gondii* growth, while glucose and fatty acids (oleic acids) did not affect parasite growth. Although inhibition of host cholesterol synthesis and uptake resulted in strong growth inhibition in RH, growth of the ME49 strain was only partially suppressed. Importantly, we showed that lower levels of LDL-derived host cholesterol induced bradyzoite conversion of the ME49 strain in CHO cells. This observation was also confirmed in HFF cells (data not shown). While there was no significant difference in parasite growth after 44 h of incubation in medium containing LPDS or FBS, BAG1 expression levels increased after 72 h incubation in LPDS. These results suggest that a gradual reduction in parasite growth occurred following BAG1 expression. Our findings are noteworthy because they suggest that starvation of specific nutrients is a trigger for parasite stage conversion. The ME49 strain may adjust to host cholesterol starvation by converting into quiescent encysted bradyzoites to reduce its cholesterol consumption. Because the RH strain is considered to be less cystogenic than the ME49 strain, lack of this nutrient may be lethal to this parasite. These data suggest different cholesterol metabolism exists between RH and ME49 strains.

Although tissue cysts containing *T. gondii* bradyzoites are found in multiple organs of the host, there appears to be preferential development of the cysts within muscular and neural tissue [19], suggesting that host cell type plays a role in this process. Primary skeletal muscle cells trigger bradyzoite conversion in the absence of exogenous stress factors [16]. Similarly, the RH strain can also differentiate spontaneously into bradyzoites in primary skeletal muscle cells [20]. In addition, *T. gondii* tachyzoite infection of skeletal muscle cells led to an increase in lipid droplet numbers [8]. The lipid droplets were in direct contact with the PV membrane, indicating that the lipid droplets were recruited for delivery inside the PV in *T. gondii*-infected skeletal muscle cells [8]. These results suggest that lipoprotein uptake by skeletal muscle cells plays a role in parasite stage conversion. In the central nervous system, cholesterol production by glial cells and its delivery via apoE-containing lipoprotein plays a crucial role in brain function [21]. Indeed, massive synaptogenesis requires large amounts of cholesterol [22]. After neural cells are infected with *T. gondii*, the parasite may compete with the host cell for cholesterol acquisition, resulting in tachyzoite to bradyzoite conversion.

## Conclusions

Our results indicate that *T. gondii* utilizes host cholesterol derived from LDL in CHO cells and that bradyzoite conversion occurs in the absence of LDL in these cells. Our data and data from previous studies suggest that host cell type plays a role in LDL-mediated stage conversion in *T. gondii*. Hence, it is important to consider the contribution that host cell factor(s) play in stage conversion. Elucidation of the molecular mechanisms underlying cholesterol metabolism in parasite stage conversion in different host cell types forms the basis of our future studies.

## Abbreviations

LDL: Low-density lipoprotein; LPDS: Lipoprotein deficient serum; PV: Parasitophorous vacuole; CE: Cholesterol esters; ACAT: Acyl-CoA: Cholesterol acyltransferase; TAG: Triacylglycerol; DGAT: Acyl-CoA: diacylglycerol acyltransferase; IFN-γ: Interferon-gamma; HFF: Human foreskin fibroblast; FBS: Fetal bovine serum; CHO: Chinese hamster ovary; LPDS: Lipoprotein deficient serum; PBS++: PBS containing 1 mM CaCl_2_ and MgCl_2_; BSA: Bovine serum albumin.

## Competing interests

The authors declare that they have no competing interests.

## Authors’ contributions

YN and FI designed the study and prepared the manuscript. YN performed the experiments. YN and FI analyzed the results. Both authors have read and approved the final manuscript.
